# Association between genotype and drug resistance profiles of *Mycobacterium tuberculosis* strains circulating in China in a national drug resistance survey

**DOI:** 10.1371/journal.pone.0174197

**Published:** 2017-03-23

**Authors:** Yang Zhou, Susan van den Hof, Shengfen Wang, Yu Pang, Bing Zhao, Hui Xia, Richard Anthony, Xichao Ou, Qiang Li, Yang Zheng, Yuanyuan Song, Yanlin Zhao, Dick van Soolingen

**Affiliations:** 1 Chinese Centre for Disease Control and Prevention, Changping district, Beijing, China, P.R; 2 KNCV Tuberculosis Foundation, CC The Hague, The Netherlands; 3 Department of Global Health, Amsterdam Medical Center, Pietersbergweg 17, BM Amsterdam, The Netherlands; 4 National Institute for Public Health and the Environment, the Netherlands,BA Bilthoven, The Netherlands; St Petersburg Pasteur Institute, RUSSIAN FEDERATION

## Abstract

We describe the population structure of a representative collection of 3,133 *Mycobacterium tuberculosis* isolates, collected within the framework of a national resistance survey from 2007 in China. Genotyping data indicate that the epidemic strains in China can be divided into seven major complexes, of which 92% belonged to the East Asian (mainly Beijing strains) or the Euro-American lineage. The epidemic Beijing strains in China are closely related to the Beijing B0/W148 strain earlier described in Russia and a large cluster of these strains has spread national wide. The density of Beijing strains is high in the whole of China (average 70%), but the highest prevalence was found North of the Yellow river. The Euro-American lineage consists of three sublineages (sublineage_1, 2, and 3) and is more prevalent in the South. Beijing lineage showed the highest cluster rate of 48% and a significantly higher level of resistance to rifampicin (14%, p<0.001), ethambutol (9%, p = 0.001), and ofloxacin (5%, p = 0.011). Within the Euro-American Lineage, sublineage_3 revealed the highest cluster rate (28%) and presented a significantly elevated level of resistance to streptomycin (44%, p<0.001). Our findings suggest that standardised treatment in this region may have contributed to the successful spread of certain strains: sublineage_3 in the Euro-American lineage may have thrived when streptomycin was used without rifampicin for treatment, while later under DOTS based treatment, in which rifampicin plays a key role, Beijing lineage appears to be spreading.

## Introduction

Among other countries in the world, China has the third highest prevalence of tuberculosis (TB) and the second largest number of multidrug resistant tuberculosis cases (MDR-TB) in the world, with a WHO estimated burden of 1.2 million TB and 52,000 MDR-TB cases in 2014[1]. Despite this heavy burden of tuberculosis, there is a relatively underdeveloped knowledge on the key elements of the causative *Mycobacterium tuberculosis* epidemic strains, also in relation to the large problem of drug resistance in China.

The global population of *M*. *tuberculosis* bacteria has been divided into seven major lineages based on genomic large sequence polymorphisms or single nucleotide polymorphism [2,3]. Distribution of lineages was found to be phylogeograghically and host associated [2]. Using spoligotyping, the Beijing genotype was first described in 1995 [4]. Later studies have reported that ‘Lineage 2’, also referred to as ‘East Asian lineage’ and almost entirely consisting of Beijing genotype strains, is predominant in the Far East and overall causes more than 60% of the tuberculosis cases in this region [2,5].

Though whole genome sequencing has shown the highest resolution in studying transmission and could provide the most detailed view of the genotype diversity, lack of an international standardization, and shared approaches in universal software for analysis and database tools, and the financial burden have so far limited its wide implementation, especially in a high burden environment [6]. On the other hand, almost all Beijing strains share the same spoligotyping pattern, which makes spoligotyping an unsuitable tool to study the inner-population genetic diversity of the Beijing strains. For the non-Beijing *M*. *tuberculosis* strains in China, primarily from the Euro-American lineage, spoligotyping has been found to be unreliable for identification of this lineage, resulting in confusing and inaccurate assignment of strains [7,8]. Thus the introduction of a more powerful and yet robust genotyping method to investigate the population structure of *M*. *tuberculosis* in China is vital. Variable-number-tandem-repeats (VNTR units) are microsatellite-like sequences in the *M*. *tuberculosis* genome and are widely used to study the population structure and transmission of this microorganism. The 24 loci VNTR typing serves as an international gold standard and has been implemented in many molecular epidemiological studies on tuberculosis [9]. It has shown robustness to investigate the population structure of *M*. *tuberculosis* [9–12]. However, it has so far not been widely applied in China for analysis of the molecular epidemiology of tuberculosis. Moreover, most data that is available is not based on nationwide, representative sampling.

Genetic lineages have been reported to be associated with different levels of pathogenicity and resistance [13–15]. Insights into the genetic background of bacteria can help to understand *M*. *tuberculosis* prevalence and transmission [16]. We therefore performed the internationally standardized 24 loci VNTR typing on more than three thousand *M*. *tuberculosis* samples collected throughout the country in the 2007 nationwide drug resistance survey, to investigate the population structure of this bacterium, and the relationship between the epidemic *M*. *tuberculosis* strains and drug resistant tuberculosis in China.

## Materials and methods

### Strain collection

All strains were collected within the framework of the first nationwide drug resistance baseline survey in 2007[17]. Briefly, a cluster-randomized sampling method was used to obtain a representative sampling of patients. Seventy sampling sites (counties) were selected in proportion with the number of reported smear positive cases in 2004 and 2005 in each province, and at least one site in each province was included. The sample size was determined to represent the estimated rifampicin resistance prevalence (6% in new and 16% in retreated patients). At each site, 51 new patients and 17 retreated patients were recruited if the smear result was positive during a period of nine months from April to December in 2007, or until the required number was met.

### Laboratory methods

The proportional method on Löwenstein-Jensen medium was applied to test the susceptibility to four first-line drugs (rifampicin, isoniazid, streptomycin, and ethambutol) and two second-line drugs (kanamycin and ofloxacin) according to the WHO guidelines. Kanamycin was chosen over amikacin and capreomycin due to the availability in clinical practice, and because of the critical concentration for amikacin on solid media of proportional method not available yet by 2008 when the drug susceptibility was tested. Para-nitro benzoic acid (PNB) and Thiophene-2carboxylic acid hydrazide (TCH) medium tubes were used for identification of the species *M*. *tuberculosis*. Spoligotyping and standardized 24 loci VNTR typing was carried out according to the protocol [9,18]. Capillary electrophoresis was used to determine the length of the PCR fragments for MIRU-VNTR test. For samples of which amplification failed at one or more loci, the whole procedure was repeated at least twice. Samples that failed to produce a band at any locus, or showed more than one band at any locus, were excluded from the subsequent analysis.

### Data analysis

Results of spoligotyping were compared to the SITVIT2 database (http://www.pasteur-guadeloupe.fr:8081/SITVIT_ONLINE/) to assign strains to clades [19]. A cluster was defined as two or more strains with identical VNTR patterns. All the VNTR patterns were compared with that of the reference strains in the MIRU-VNTRplus website database (http://www.miru-vntrplus.org/MIRU/index.faces) to identify their lineages by similarity and assigned a MLVA code for each pattern if possible [20]. Similarity between VNTR patterns and reference strains was measured by categorical distance which was defined by dividing the number of loci differing between the two by the total number of MIRU-VNTR loci. When the genotype was not represented in the reference database, spoligotyping was used to assigned the genotype to a known lineage. Cluster rate was defined as *n*_*c*_/*N*, in which N is the total number of strains in the sample, and *n*_*c*_ is the total number of clustered strains. BioNumeric 5.0 was used to produce a minimum spanning tree on basis of the 24 loci VNTR typing results with categorical method. The criterion for categorizing a complex was set to at most differences at any three of these 24 loci to best reflect the topology of the minimum spanning tree. The association between drug resistance level and genotypes were analysed with Chi-square test or Fisher exact test. Administrative boundaries were provided by the National Key Project (2014ZX10003002).

### Ethical review

This study was approved by the Tuberculosis Research Ethics Review Committee of the China CDC. All included patients signed an informed consent form.

## Results

In total, 3,929 isolates were collected from sputum smear positive tuberculosis patients with drug susceptibility test results from 70 counties in 31 provinces in the 2007 survey. VNTR typing was performed on 3,703 isolates available in our strain bank. For 143 (3.9%) isolates, multiples bands were detected at one or more loci and were excluded from further analysis. Another 427 (11.5%) isolates had one or more loci that failed PCR amplification for VNTR typing, though there may be several explanations for the failure, the exact causes were not the focus of this paper, so these strains were just excluded for the stringency of analysis. Finally, 3,133 (84.6% of the total) strains had a complete and single VNTR pattern and were included in the analyses.

There were 1,930 unique VNTR patterns and 290 clusters with identical VNTR types (VT) observed (S1 Table). In total 1,203 strains were clustered in clusters of 2 to 133 strains. Of these 290 clusters, 219 (75.5%) clusters, including the largest cluster of 133 isolates (VT007), comprised only Beijing genotype strains (894 in total). The five largest clusters (>20) were almost all Beijing strains except one strain from Manu2 and another strain from T1.

**Fig 1:**
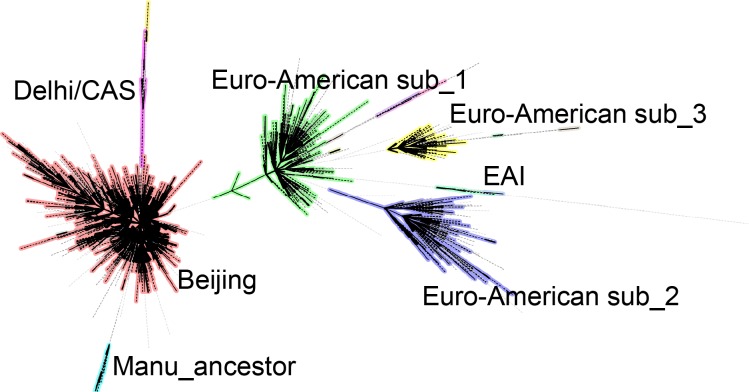
Minimum spanning tree based on 24 loci VNTR. Complexes was defined by difference at 3 or less loci of the 24 VNTR pattern. Lineage name was assigned by comparing to MIRU-VNTRplus database, or by spoligotyping for Manu_ancestor strains.

We constructed a minimum spanning tree on basis of 2,220 VNTR patterns and identified seven major complexes of more than 10 strains, each complex consisting of strains which varied maximally at 3 loci (Fig 1). Among the largest complex(n = 2208), almost all (n = 2206) of these strains was identified as Beijing lineage with an average distance to Beijing reference strains of 0.07 (corresponding to difference at 1.7 locus among 24 loci on average). Of these strains, 94% (n = 2079) was also confirmed by spoligotyping as Beijing lineage and 98% (n = 2154) if only by spoligotyping. Henceforward, the largest complex was named Beijing lineage. The following three complexes basically represented three genetically distinct sub-lineages in the Euro-American lineage, including T, S, Haarlem, LAM, and Manu clades as defined by spoligotyping and named as Euro-American sublineage_1, 2, and 3, sequentially. Compared to MIRU-VNTRplus database, Euro American sublineage_1 (n = 345) was distinguished from other lineages but similar to the New-1 strains in the MIRU-VNTRplus database. Euro American sublineage_2 (n = 225) was also different from other lineages but relatively close to S strains. Euro American sublineage_3 (n = 116) was closely related to URAL and TUR strains. The first four complexes represented 92% of the whole sample. The last three complexes were relatively small complexes, which were primarily consisted of strains close to Delhi /CAS strains (n = 21), Manu_ancestor strains (n = 14), and EAI strains (n = 10), respectively.

**Fig 2:**
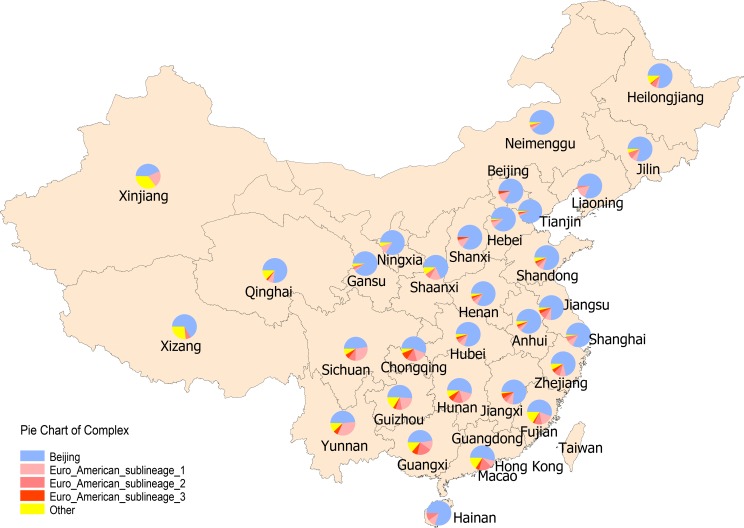
Geographic distribution of the complexes in 31 provinces in mainland of China.

Beijing lineage was predominant in all provinces and varied from 44% to 93% of the sampled strains, with an average prevalence of 70% nationwide. The highest density was observed in the provinces north of Yellow River (Fig 2) and the lowest was observed in Xinjiang province in the northwest of China. Euro-American lineage was most common in the area south of the Yellow River, with a rather even distribution of these three sublineages in this area; the highest density of these sublineages was seen in the Southwest. The north western Xinjiang province had a unique distribution of genotypes with a complex composition, as the majority of Delhi/CAS lineage strains (62%) were found in Xinjiang. It is possible that Central Asia is the origin of these Delhi/CAS strains. In contrast, Manu_ancestor and EAI lineages strains were most common in the South East coast provinces, indicating that a foreign origin from oversea is likely for these strains.

Strains with identical VNTR patterns were not geographically contained. For example, the largest cluster VT007, with the MLVA code 1048–32, comprising 133 strains, was observed in 51 (out of 70) counties and in 26 (out of 31) provinces. The largest number of VT007 strains by county was seven, and was found in two counties. The largest five clusters together, each comprising 20 or more of Beijing lineage strains and only differing at one or two VNTR loci, were retrieved from 66 sites in 28 provinces (Table 1).

**Table 1:** Five most prevalent genotypes with more than 20 strains in each genotype

MIRU-VNTR genotypes	MIRU-VNTR code*	No. of strains in cluster	MLVA code^#^	Clade in spoligotyping
VT 007	273335444432658253213423	133	1048–32	Beijing (133)
VT 028	273335444432558253213423	83	342–32	Beijing (82), Manu2(1)
VT 111	273235444432658253213423	35	204–32	Beijing (34), T1(1)
VT 064	273335444432458253213423	32	898–32	Beijing (32)
VT 048	273335444432657253213423	25	100–32	Beijing (25)

*Order of VNTR loci: MIRU4, MIRU26, MIRU40, MIRU10, MIRU16, MIRU31, Mtub04, ETRC, ETRA, Mtub30, Mtub39, QUB4156, QUB11b, Mtub21, QUB26, MIRU02, MIRU23, MIRU39, MIRU20, MIRU24, MIRU27, Mtub29, ETRB, Mtub34. The differences between clusters were shown in underlined.

#: MLVA code was given by MIRU-VNTRplus database, which is composed of a digital code for 15 loci VNTR pattern in the front and a digital code for 9 loci VNTR pattern in the end.

Significant differences in the prevalence of drug resistance were observed between the complexes (Table 2). Beijing lineage had the highest proportion of members resistant to rifampicin, isoniazid, ethambutol and ofloxacin (14%, 22%, 9% and 5%, respectively). It also revealed the highest correlation with MDR-TB rate (12%), followed by Euro-American sublineage_1 (9%), Euro-American sublineage_3 (6%) and then Euro-American sublineage_2 (4%). Euro-American sublineage_3 appeared to contain more members resistant to streptomycin (44%) or kanamycin (4.3%) than the other three complexes; 31% of Euro-American sublineage_3 strains were streptomycin mono-resistant, which was more than that in any other major complex. Streptomycin mono resistance varied from 11% to 22% in the other complexes tested.

**Table 2:** Drug resistance per complex.

Drug*	Beijing lineage(n = 2208)	Euro-American sublineage_1 (n = 345)	Euro-American sublineage_2 (n = 225)	Euro-American sublineage_3 (n = 116)	P-value^#^
	number of strains (%, 95%CI)				
Pan-susceptible	1389 (62.9,60.9–64.9)	221 (64.1,58.8–69.1)	131 (58.2,51.5–64.7)	56 (48.3,38.9–57.8)	0.006
Any resistance					
Isoniazid	476 (21.6,19.9–23.3)	61 (17.7,13.8–22.1)	38 (16.9,12.2–22.4)	18 (15.5,9.5–23.4)	0.08
Streptomycin	602 (27.3,25.4–29.2)	85 (24.6,20.2–29.5)	75 (33.3,27.2–39.9)	51 (44.0,34.8–53.5)	<0.001
Ethambutol	195 (8.8,7.7–10.1)	18 (5.2,3.1–8.1)	11 (4.9,2.5–8.6)	4 (3.4,0.9–8.6)	0.007
Rifampicin	304 (13.8,12.4–15.3)	32 (9.3,6.4–12.8)	13 (5.8,3.1–9.7)	8 (6.9,3.0–13.1)	<0.001
Kanamycin	45 (2.0,1.5–2.7)	6 (1.7,0.6–3.7)	2 (0.9,0.1–3.2)	5 (4.3,1.4–9.8)	0.195
Ofloxacin	107 (4.8,4.0–5.8)	9 (2.6,1.2–4.9)	6 (2.7,1.0–5.7)	0 (0.0,0.0–3.1)	0.013
MDR	266 (12.0,10.7–13.5)	31 (9.0,6.2–12.5)	10 (4.4,2.2–8.0)	7 (6.0,2.5–12.0)	<0.001
XDR	19 (0.9,0.5–1.3)	1 (0.3,0.0–1.6)	1 (0.4,0.0–2.5)	0 (0.0,0.0–3.1)	0.464

* pan-susceptible means susceptible to all six drugs tested; MDR, resistant to at least rifampicin and isoniazid; XDR were also MDR strains and plus resistance to ofloxacin and streptomycin/kanamycin.

^#^ P-value refers to the comparison of the resistance level in each complex and was calculated with Chisq-test or Fisher exact test.

We also compared the proportion of strains with resistance to streptomycin and/or rifampicin in each complex (Table 3). Interestingly, we observed no significant difference in rifampicin resistance in the absence of streptomycin resistance among these four largest complexes (p = 0.411), although we again observed a significant difference in streptomycin resistance in rifampicin sensitive strains of the different complexes, with the highest proportion of 41% in Euro-American sublineage_3 and the lowest of 18% in Beijing lineage(p<0.001). On the contrary, resistance to both streptomycin and rifampicin was highest in Beijing lineage (9%) and lowest in Euro-American sublineage_3 (3%, p = 0.008). Accordingly, MDR strains in Beijing lineage showed higher resistance level to streptomycin (74%, 196/266) than non-MDR strains in Beijing lineage (21%, 406/1942, p<0.001); while this difference was not significant in Euro-American sublineage 3 (57%, 4/7 and 43%, 47/109, respectively; p = 0.698).

**Table 3:** Streptomycin and rifampicin resistance per complex.

Drug resistance*	Beijing lineage (n = 2208)	Euro-American sublineage_1 (n = 345)	Euro-American sublineage_2 (n = 225)	Euro-American sublineage_3 (n = 116)	P-value^#^
	Number of resistant strains(%,95%CI)				
Susceptible	1510 (68.4%,68.1–68.7)	243 (70.4%,69.9–71.1)	145 (64.4%,64.0–65.2)	61 (52.6%,51.7–53.4)	<0.001
SM-R/RIF-S-	394 (17.8%,17.6–18.2)	70 (20.3%,19.7–20.9)	67 (29.8%,29.3–30.5)	47 (40.5%,39.7–41.4)	<0.001
SM-S/RIF-R	96 (4.3%,4.1–4.7)	17 (4.9%,4.3–5.6)	5 (2.2%,1.8–3.0)	4 (3.4%,2.6–4.3)	0.411
SM-R/RIF-R	208 (9.4%,9.1–9.8)	15 (4.3%,3.8–5.0)	8 (3.6%,3.1–4.3)	4 (3.4%,2.6–4.3)	0.008

* susceptible strains were susceptible to both streptomycin (SM) and rifampicin (RIF); SM-R/RIF-S strains were resistant to streptomycin but susceptible to rifampicin; SM-S/RIF-R strains were resistant to rifampicin but susceptible to streptomycin; SM-R/RIF-R strains were resistant to both streptomycin and rifampicin.

^#^ P-value refers to the comparison of the resistance level in each complex and was calculated with Chisq-test or Fisher exact test.

Complexes also revealed different degrees in clustering (Table 4). The overall cluster rate was 38%. Beijing lineage showed the highest cluster rate (48%, p<0.001). Within Euro-American lineage, the three sublineages also differed significantly in degree of clustering: Euro-American sublineage_3 had the highest clustering proportion (28%), while Euro-American sublineage_2 had the lowest (10%).

**Table 4:** Cluster rate in different genotypes.

Genotypes	No. of cases	No. of clustered cases	Cluster rate	
			%	95% CI
Beijing lineage	2208	1061	48	46.0–50.2
Euro-American sublineage_1	345	65	19	14.9–23.
Euro-American sublineage_2	225	23	10	6.6–14.9
Euro-American sublineage_3	116	33	28	20.5–37.6
Other complexes	239	21	9	5.5–13.1

## Discussion

Previous studies have shown that Beijing lineage was predominant in China, followed by Euro-American lineage [5,21,22]. Whole genome sequencing and MIRU-VNTR was also utilized in several studies looking into the phylogeny and transmission of M. tuberculosis in China, but at a smaller scale and with different sampling schemes [23–25]. To investigate the epidemic strains in China and the relation between genotypes and drug resistance, we performed the standardized method 24 loci MIRU-VNTR on the most representative yet largest sample so far to determine the population structure of *M*. *tuberculosis* at a nationwide scale. We demonstrate there are seven major genotype complexes circulating in China based on VNTR and spoligotyping, which are all members of five major lineages: the largest complex Beijing lineage; three sublineages or complexes within Euro American lineage, and three relatively small complex representing Delhi/CAS, Manu_ancestor, and EAI lineage, respectively [19,26].

As previously reported, the Beijing lineage is the predominant lineage in China, followed by Euro American lineage [5,9]. Our study indicates that these two major lineages are wide spread nationwide in China, but revealed differences in their distribution. With some exceptions, differences in the prevalence of these two lineages roughly followed the Yellow River. This is interesting from the perspective of the dynamics of the TB epidemic in China. One possible underlying reason is that Beijing lineage in China originated in the North, while the Euro American lineage originated in the South. Previous studies reported that the high genetic diversity of Beijing genotypes in Japan is consistent with their likely origin in this region, indicating a more ancient common ancestor for the Beijing genotype strains in North-Eastern Asia [27,28]. Conversely, some studies have detected Beijing (like) strains with a full spoligotyping pattern and a RD105 deletion or “extended” RD105 deletion in Southwest of China, which would be consistent with the common ancestor of the currently expanding Beijing strains originating from this region [23,29]. This hypothesis is also supported by the study by Luo *et al*. using whole genome sequencing and the study by Yin et al. using 24 loci MIRU-VNTR [23,30]. However, yet another study suggests that the deletion of the 1–34 spacers in spoligotyping may have happened before the RD105 deletion [31]. This would make the Southwest China “ancestor” type of Beijing family unlikely to be the real ancestor and instead implies the RD105 region has been independently lost at least twice. Nonetheless, a study with a global sample of thousands of Beijing strain supports the Far East origin of Beijing genotype [32]. All these results together indicate that Beijing genotype now present globally may indeed originate from East Asia, but the detailed history of divergence and spread needs to be further addressed.

Nearly 90% of all rifampicin resistant strains are multidrug resistant [17]. A previous report has shown that Beijing strains isolated from MDR-TB cases were associated with streptomycin resistance [33]. So we further looked into the interactions of these two drugs with genotypes. We observed a higher level of resistance to rifampicin and streptomycin in Beijing lineage and Euro-American sublineage_3, respectively. However, the proportion of strains resistant to both drugs were significantly higher in Beijing lineage than in Euro-American sublineage_3. Streptomycin was the first antibiotic used to treat tuberculosis and was first introduced into China in 1947[34,35]. Unfortunately, after initial clinical improvement in the first few months, drug resistance in patients treated with streptomycin as a mono therapy frequently developed [34]. Combined treatment by four drugs, including rifampicin, isoniazid, pyrazinamide and ethambutol was developed in 1970s, which increased the cure rate and reduced the relapse rate[36,37]. However, MDR-TB as a consequence of this treatment regimen was recognized in the 1990s and prompted the declaration of the WHO on tuberculosis being a global health emergency. Standardised treatment is a strong selective pressure and we assume that the population structure of the prevalent *M*. *tuberculosis* strains has been influenced by the emergence of drug resistance. When streptomycin was first introduced, it appears to have resulted in the expansion of some lineages, Euro-American sublineag_3 for example. This would imply that Euro-American sublineage_3 has an increased potential to develop streptomycin resistance, while maintaining virulence and the ability to spread, which is also consistent with the higher cluster rate of Euro-American sublineage_3 compared to other sub-lineages, as these streptomycin resistant strains remain present in our current sample and seem to continue to spread. Later on, when treatment by rifampicin was introduced, this lineage, especially the part resistant to streptomycin in this complex, appears to have been less successful at generating resistance to the new drugs than another complex, Beijing lineage in this case. The latter complex showed a higher capability to escape the antimicrobial activity of rifampicin and restore virulence when compared to other complexes, even with already acquired resistance to streptomycin as shown in our dataset. This is in line with the recently observed remarkable tolerance of Beijing genotype strains to rifampicin, as shown in advanced proteomics experiments[38,39]. Potentially, this is a very important observation, as it would imply that specific lineages were better at adapting to specific treatment regimens. Especially, according to our data, MDR Beijing strains appears to have a higher potential to be resistant to streptomycin than non MDR Beijing strains and other genotypes, which may be important when treat previously failed MDR-TB patients infected with Beijing strains, since three out of the five antibiotics in the standard regime for retreatment patients were likely to be ineffective [40]. However, this issue needs to be further addressed and ideally explained by *in vitro* and *in vivo* experiments.

We found several large clusters of strains with identical VNTR patterns spreading in wide geographic regions. Although we have not investigated the actual epidemiological links between these clustered cases, it is reasonable to infer that such large scale links will be rather hard to establish. We assume these strains with identical VNTR pattern are genetically closely related and successful strains which are able to spread in diverse locations. In support of this, the VT048 signature, which belongs to the most prevalent Beijing B0/W148 cluster in Northwest Russia and East Europe, corresponding to the MLVA code 100–32 in the MIRU-VNTRplus database, was also the fifth largest cluster in our sample [41,42]. This genotype has been reported circulating in China in previous studies [23,30]. Moreover, in total 688(21%) strains in our sample, including the other four largest clusters, differed only at one or two loci from this genotype, indicating the epidemic Beijing strains in China are genetically very closely related to the Beijing B0/W148 strains in Russia. Beijing B0/W148 strains were reported to show stronger association with MDR-TB and higher transmissibility [43–46]. Accordingly, these regions with a high prevalence of Beijing B0/W148 strains were also reported to have a heavy MDR-TB burden, indicating this closely related group of strains appears to be able to successfully generate resistance to the current standard therapy while they remain transmissible in certain settings [1,46]. Searching through literature, we found these epidemic strains in China were previously also reported in Europe and North Africa but at a low prevalence, suggesting these clusters are globally wide spread but with different levels of adaptation in different areas [47,48].

From this study we have shown strong evidence of association between genotypes and drug resistance with the largest sample ever been collected in China so far. Meanwhile, the finding that there are different distributions of drug resistance in different bacterial genotypes also raised the probability that standardised, possibly sub-optimal treatment regimens may result in the selection of specific strains, as indicated by previous studies [49,50]. Though some questions, such as the origin and driving force of the spread of the Beijing genotype, as well as the circulation of some extremely large clusters in China (and beyond), still remain to be elucidated by further investigation with appropriate methods, whole genome sequencing for example, an awareness of the differences in the biology and epidemiology between different *M*. *tuberculosis* (sub) lineages will help to understand the nature of the circulating strains and deal with this ancient, yet continuously developing threat to public health.

## Supporting information

S1 Table (XLSX)
